# Amplicon-based long-read sequencing for accurate *CYP2D6* gene deletion and duplication detection using *CYP2D7* as a reference gene

**DOI:** 10.1038/s41397-026-00429-x

**Published:** 2026-08-26

**Authors:** Kiflu Gebremicael Tesfamicael, Michael Musker, David L. Adelson, Martin David Lewis

**Affiliations:** 1 Lifelong Health, South Australian Health and Medical Research Institute (SAHMRI), Adelaide, SA Australia; 2 School of Biological Sciences, Adelaide University, Adelaide, SA Australia; 3 South Australian Research and Development Institute (SARDI), Department of Primary Industries and Regions, Adelaide, Australia; 4 School of Nursing and Midwifery, College of Health, Adelaide University, Adelaide, SA Australia; 5 South Australian Museum, Adelaide, Australia

**Keywords:** Sequencing, Predictive markers

## Abstract

We developed an amplicon-based long-read sequencing approach that enables simultaneous detection of *CYP2D6* single-nucleotide variants (SNVs) and copy-number variations (CNVs) in a single reaction. The SNVs of the two pseudogenes, *CYP2D7* and *CYP2D8*, were also comprehensively characterized. *CYP2D6 CNVs* were inferred using *CYP2D7* as a reference gene, integrating read-depth measurements with allele-frequency (AF) ratios. The entire *CYP2D* locus ( ~ 30.2Kb) and the three homologous genes separately (6.3–6.6Kb) were sequenced using Oxford Nanopore sequencing. Our analysis identified 21 *CYP2D7* and 17 *CYP2D8* unique haplotypes, and 57% of the SNVs detected in the pseudogenes had nucleotides corresponding to *CYP2D6* reference nucleotides. A total of 36 variants found within the pseudogenes have also been annotated as functional variants in the *CYP2D6* gene. The combined *CYP2D6*:*CYP2D7* read-count ratio and reference-to-alternative alleles AF ratio accurately predicted *CYP2D6* CNV status. This method is simple and rapid, making it potentially suitable for clinical implementation of pharmacogenomics.

## Introduction

*CYP2D6* is a highly complex gene crucial for drug metabolism and brain function [1]. It is involved in the metabolism of over 25% of all therapeutic [2] and 69% of psychiatric drugs [3], and influences neurotransmitter production and brain activity [4, 5]. Despite its importance, testing for *CYP2D6* variants is not yet a standard practice in clinical settings [6]. This is mainly due to the difficulty of *CYP2D6* genotyping, which is attributed to its high polymorphism level and homologous pseudogenes [7].

The *CYP2D6* gene harbours over 160 unique haplotypes comprising 354 SNPs, 36 indels, a full gene deletion, duplications (up to 12 copies), and hybrid variants [8]. *CYP2D6* variants alter gene function and, consequently, drug metabolism and drug response [9]. Therefore, precise prediction of *CYP2D6* function depends entirely on accurate detection of its variants, phasing, and phenotype translation. In addition, the *CYP2D6* gene is flanked by two highly homologous pseudogenes, such as *CYP2D7* and *CYP2D8*, which share 95–97% sequence similarity with *CYP2D6* [10]. This can cause misalignments and increase the possibility of genotyping errors [11]. Furthermore, variants in these pseudogenes can interfere with *CYP2D6* genotyping [12]. Overall, the *CYP2D6* gene is complex, and accurate genotyping of this gene has been substantially difficult for both research and clinical applications, primarily using microarray-based techniques and short-read Next-Generation-Sequencing (NGS) technologies [12, 13].

Microarray-based and short-read NGS methods are limited for genotyping complex genes, such as *CYP2D6*, resulting in a high rate of genotyping errors due to misalignment and phasing difficulties [13]. Most genotyping communities used microarray-based techniques to genotype *CYP2D6*, but these methods can identify only a small percentage of known *CYP2D6* variants; for example, the Axiom_UKB assay detects only 20% of known *CYP2D6* star alleles [14]. Recent advancements in long-read sequencing (LRS), such as PacBio and Nanopore sequencing, have enabled the sequencing of the entire *CYP2D6* gene, including the promoter region (~ 6.6 kb) [12, 15–17]. The long read generated without fragmentation enabled direct single-nucleotide variation (SNV) calling with accurate phasing [12]. However, identifying copy number variations (CNVs) still requires additional assays and preparations. To date, no assay or method can detect all *CYP2D6* variants in a single run [18]. It requires multiple preparations and analyses, along with the combination of several different assay types targeting different variants. These challenges reduce the feasibility of routine clinical *CYP2D6* testing and represent a significant barrier to its broader adoption.

The purpose of this study was to develop an improved methodology capable of detecting *CYP2D6* SNVs and CNVs in a single assay. We characterised the SNVs of the two homologous *CYP2D* pseudogenes to assess potential misalignments and genotyping errors. This would help overcome the difficulties in genotyping complex genes and make integrating PGx into clinical practice easier.

## Methods

### Samples

A total of 17 individuals with known *CYP2D6* genotypes were included in this study. DNA from these individuals was obtained from the Coriell Institute for Medical Research cell repository (Camden, NJ, USA). Samples were selected based on the availability of *CYP2D6* variants. These individuals were previously characterised for *CYP2D6* and carry wild type (*n* = 5), gene deletion (*n* = 2), duplication (*n* = 4), and *CYP2D6*::*CYP2D7* hybrids (*n* = 7), with one sample carrying an unresolved hybrid type (Table 1). One individual carries *CYP2D6* duplication and hybrids. *CYP2D6* genotypes were determined by consensus across five independent laboratories using diverse methods. *CYP2D6* CNVs and structural variants were further validated using orthogonal approaches, including quantitative copy number assays, long-range PCR, Sanger sequencing, next-generation sequencing, and SMRT sequencing. This study used commercially available, de-identified human reference materials obtained from the Coriell Institute for Medical Research; therefore, institutional ethics approval and informed consent were not required. All methods adhered to institutional guidelines and applicable regulations.

**Table 1:** List of individuals with their genotype and *CYP2D6* characteristics.

Coriell Institute cell reference	Ethnicity	*CYP2D6* gene characteristics	*CYP2D6* gene Genotype
HG00111	British	Wild	*3/*3
NA10005	Black/African America	Wild	*17/*29
NA10181	Ashkenazi/White	Wild	*1/*41
NA19789	Mexican Ancestry	Wild	*1/*1
NA23877	NA	Wild	*15/*41
NA08873	African American	Partial deletion	*5/*17
NA19317	Luhya	Full deletion	*5/*5
NA17164	African American	Partial duplication	*4×2/*41
NA17244	White	Full duplication and *CYP2D6*::*CYP2D7* hybrid	*2×2/*4×2 (+ hybrid)
NA17454	Mexican American	Full duplication	*1×2/*2×2
NA18526	Han Chinese	Partial duplication and CYP2D6::*CYP2D7* hybrid	*1/*36×2 + *10
HG00463	Southern Han Chinese	*CYP2D6::CYP2D7* hybrid	*36 + *10/*36 + *10
NA12154	Utah/Mormon/White	*CYP2D6::CYP2D7* hybrid	(*68) +*4/*33
NA17209	White	*CYP2D6::CYP2D7* hybrid	*1/*4 + *36
NA17287	Italian/white	*CYP2D6::CYP2D7* hybrid	*1/*83
NA18540	Han Chinese	*CYP2D6::CYP2D7* hybrid	(*36 + )10/*41
NA18564	Han Chinese	*CYP2D6::CYP2D7* hybrid	*2 A/*36 + *10

*Wild *CYP2D6* gene characteristics indicate the absence of CNV/SV.

### Experimental design

We designed a unique primer targeting *CYP2D6*, *CYP2D7*, *CYP2D8* and the entire *CYP2D* locus (Table 2). Triplex PCR was performed to amplify the *CYP2D6*, *CYP2D7*, and *CYP2D8* genes using primers designed to target them. Primers were designed using the Primer3 program [19] to generate amplicons of similar sizes (6.3Kb–6.6Kb), concentrations and annealing temperatures, ensuring uniform amplification efficiency across all target genes. Primers’ specificity was confirmed using Primer-Blast searches and validated in silico using the web-based Net Primer [20]. After confirming successful amplification and checking for primer-dimer formation, primers were pooled at equimolar concentrations for the triplex PCR. The entire *CYP2D6-2D7-2D8* locus, spanning up to 30.2Kb, was amplified using three overlapping primers listed in Table 2. The overlapping amplicons ranged in size from 8.3–11Kb. To amplify the entire *CYP2D* locus, three singleplex PCRs for the overlapping regions were performed for each sample, confirmed on gel electrophoresis, and then pooled equimolarly for each sample.

**Table 2:** Primer sequences for *CYP2D6*, *CYP2D7*, *CYP2D8*, *CYP2D6-2D7* hybrids and overlapping read for the entire *CYP2D* loci.

Target region	Primers	Amplicon size (kb)	Type of PCR
*CYP2D6*	F: ATGGCAGCTGCCATACAATCCACCTG	6.6	Triplex
	R: ACTGAGCCCTGGGAGGTAGGTAG		
*CYP2D7*	F: GTGGCTGCCCCATGTCATGTATTGG	6.3	
	R: GCCCTGGGAGGTAGCCCTGGCATA		
*CYP2D8*	F: TCATTCAGCTGAGGCCAGTTTGGGA	6.6	
	R: CTGGTGGTCACGTGAAGTCAGGAGT		
Entire CYP2D	F: GGGGATGGGGTCTTGCTAGGTCTC	9	Singleplex
(*CYP2D6* region)	R: CTGGGAGGTAGGTAGCCCTGGCATA		
Entire CYP2D	R: CGGCAGTGGTCAGCTAATGAC	11.5	
(*CYP2D7* region)	F: TCATTCAGCTGAGGCCAGTTTGGGA		
Entire CYP2D	F: GCCACCATGTAACAAGCACCCAGAC	8.3	
(*CYP2D8* region)	R: CCACATTCAGCATTGGACGGGTGTC		
*CYP26-2D7 Hybrids*	F: 5′ ATGGCAGCTGCCATACAATCCACCTG	5.47	
	R: 5’ GCCCTGGGAGGTAGCCCTGGCATA		

### Long-range PCR

Both triplex and singleplex long-range (XL) PCR were performed using Takara v2 polymerase according to the manufacturer’s instructions. A total of 20 μL reaction volume containing ~80 ng of genomic DNA, 400 nM gene-specific primers, and 7 μL of Takara kit mix was used. The reaction mix was run on a thermocycler for 1 min at 94 °C, followed by 30 cycles of 10 s at 98 °C and 8 min at 68 °C, and a final extension of 10 min at 68 °C. The successful amplification of the target genes was confirmed on 1% (w/v) agarose gel electrophoresis, and the amplicons were cleaned using AMPure XP magnetic beads (0.8×). The amplicon concentration was measured using a Qubit fluorometer, and equal concentrations were prepared for each sample.

The Nanopore sequencing library was prepared using the Native Barcoding Kit 24 (SQK-NBD112.24) according to the manufacturer’s instructions. PCR amplicons from individual samples (856 ng DNA amplicons) were subjected to end-repair using NEBNext Ultra II End repair/dA-tailing Module (third-party reagent) and barcoded using Native barcodes (NB01-24). Barcoded samples were pooled, and Oxford Nanopore Technologies (ONT) sequencing adaptors were ligated. Samples were cleaned and purified using Long Fragment Buffer (LFB) and AMPure Beads (AXP). After purification, 85.5 ng (∼20 fmoles) of the prepared library were loaded into the R9.4.1 flow cell (FLO-MIN106) and sequenced for 40 h on the MinION (Oxford Nanopore Technologies). The raw MinION sequencing output was stored in Fast5 format.

### Variant calling

Basecalling, converting the Fast5 file to Fastq files, was performed using Guppy Basecaller version 6.4.6 (ONT, Oxford, UK). The Guppy barcoder program was used to demultiplex and trim adaptors and barcodes from the Fastq files. Sequencing reads (Fastq files) with read lengths of less than 6 kb and read qualities of less than ten were filtered out using NanoFilt [21]. Filtered sequence reads were aligned to the reference genome (hg38) using Minmap2 [22] and long read aligner (lra) [23]. The two long-read aligning software were compared for *CYP2D6* CNV detection and variant calling. The aligners’ performance was mainly evaluated based on the specificity of read alignment, i.e., lower multiple read alignments. This was achieved by comparing the numbers of raw reads counted with FastQC and unfiltered mapped reads counted with BamTools [24]. Unmapped reads were removed using SAMtools [25] to obtain high-quality reads aligned to each gene. We included reads with mapping quality greater than 30 and mapping alignment span (rlen) greater than 6 kb. For variant calling, we used and compared three long-read variant callers: PEPPER-Margin-DeepVariant [26], Clair3 [27], and NanoCaller [28]. For using PEPPER-Margin-DeepVariant, the filtered aligned files were downsampled to ~100X using Picard Toolkit version 3.0.0 [29].

### *CYP2D6* copy number

The *CYP2D7* gene was used as a reference gene to determine the *CYP2D6* CNV. Firstly, the total number of filtered sequence reads that uniquely aligned to the *CYP2D6* gene (GRCh38p.14; Chr22:42126000-42132400) or the *CYP2D7* gene (GRCh38p.14; Chr22:42139000-42145500) was counted using BamTools [24]. The read count ratio between the two genes was compared, and the *CYP2D6* CNV was calculated. This calculation assumes no CNV (CN = 2) for the *CYP2D7* gene. Therefore, for individuals without a CNV (wild *CYP2D6* gene), a 1:1 read ratio would be expected between these two genes. Conversely, individuals exhibiting a full *CYP2D6* duplication (CN = 4) would display approximately twice the number of reads compared to *CYP2D7*, yielding a read ratio of 2:1. Full duplication or deletion refers to the duplication or deletion of both haplotypes of the gene. In contrast, partial duplication or deletion refers to the duplication or deletion of only one haplotype of the gene. In cases of partial deletion (CN = 1), the reads aligned to *CYP2D6* would amount to around half of those from *CYP2D7*, resulting in a *CYP2D6*:*CYP2D7* ratio of approximately 1:1.5. Additionally, allele frequency (AF) ratio between the reference and alternative alleles of *CYP2D6* SNPs were used to infer which haplotype of the gene was duplicated in individuals with partial CNV.

## Results

We successfully sequenced the three highly homologous *CYP2D* genes using amplicon-based long-read Nanopore sequencing for samples with and without *CYP2D6* CNV. We generated amplicon sequences of two types: the three *CYP2D* genes (*CYP2D6*, *CYP2D7*, and *CYP2D8*) and the entire *CYP2D6-2D7-2D8* locus (GRCh38p.14; Chr22:42126071-42156204. The amplicons for the three genes had relatively similar read sizes: 6.58Kb for *CYP2D6*, 6.3Kb for *CYP2D7*, and 6.6Kb for *CYP2D8*. The triplex PCR targeted these three genes and generated adequate alignable sequence reads in all individuals except for individual HG00111. Therefore, this individual was removed from the CNV analysis. A total of 115Kb reads with an average of 10.5 kb were generated across the 11 individuals included in the triplex PCR. In addition, the entire *CYP2D6-2D7-2D8* locus (~ 30.2Kb) with three overlapping PCR amplicons generated sufficient sequence reads, ranging from 733 bp–24.4 kb, with an average of 19.6 kb. For analysis, we included only high-quality reads with PHRED-scaled Q-scores averaging >30, a length of 6.0Kb, and an average read depth >150X. These filtered reads were aligned to the human genome reference (GRCh38p.14:ch22). The number of reads mapped to *CYP2D6* and *CYP2D7* genes was similar, except in individuals with CNV. However, the number of reads aligned to the *CYP2D8* gene was lower. The total number of filtered reads aligned to *CYP2D6*, *CYP2D7*, and *CYP2D8* was 33.6Kb, 28.8Kb, and 3.6Kb, respectively.

### Comparison of long-read mapping and variant calling tools

**Fig. 1:**
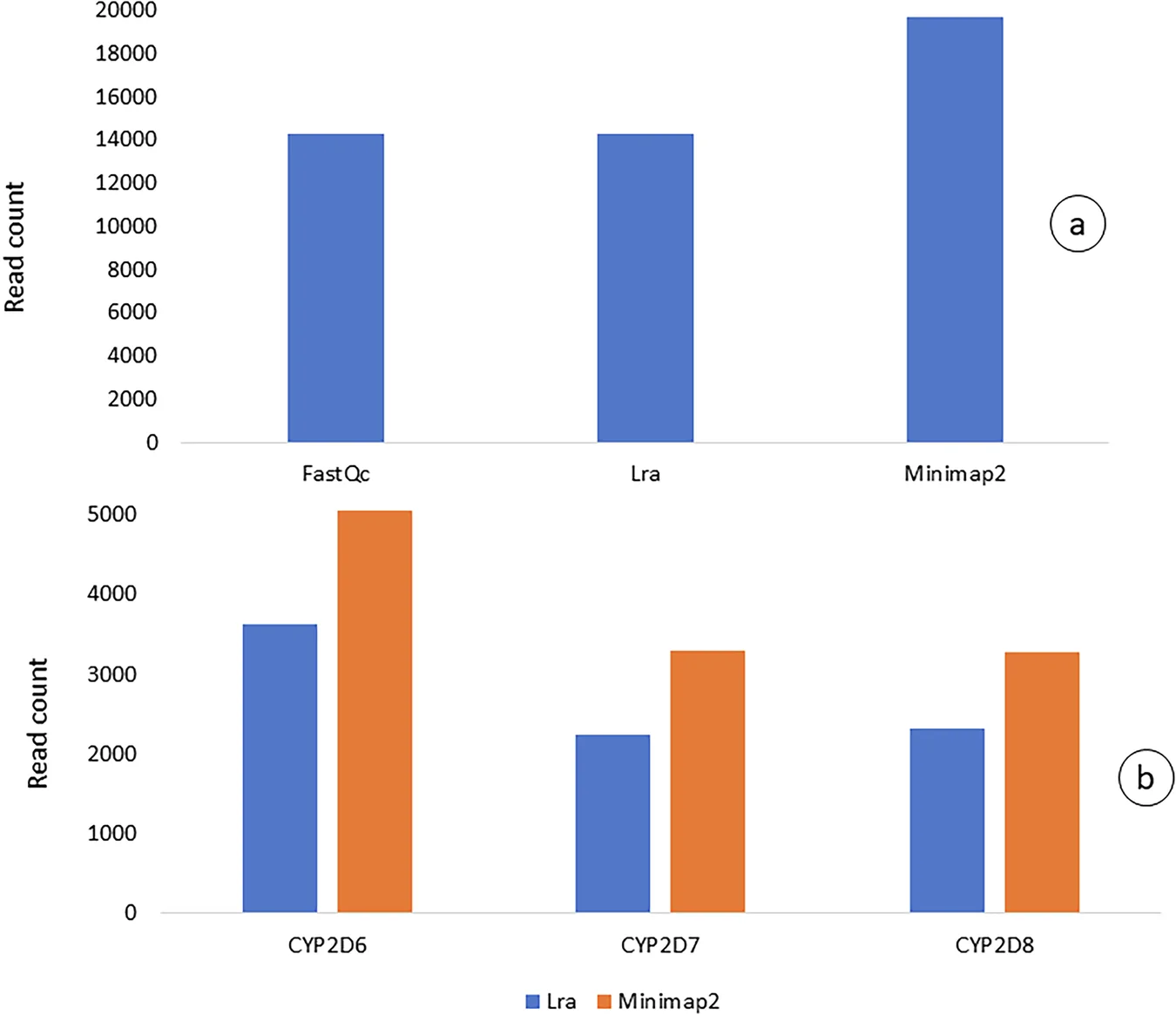
Average read counts across the 17 individuals included in this study, (**a**) average read count calculated before mapping (FastQc) and after alignment using Lra and Minimap2, (**b**) average filtered read counts aligned to *CYP2D6*, *CYP2D7* and *CYP2D8* genes.

We compared the two widely used long-read mapping tools, Lra and Minimap2. The number of raw and Lra-aligned reads was 100% identical (Fig. 1a). However, the number of aligned reads using Minimap2 was higher than the raw reads (Fig. 1b). This is due to the read’s alignment into multiple genome positions. In addition, the number of aligned reads to the *CYP2D6*, *CYP2D7*, and *CYP2D8* genes was higher with Minimap2 than with Lra (Fig. 1b). Based on our analysis, Lra has the highest alignment specificity; therefore, we used Lra to predict the *CYP2D6* gene CNV by comparing the number of reads aligned to *CYP2D6* and *CYP2D7*. In addition, these aligners were also evaluated based on variant-calling quality, compared to prior *CYP2D6* genotyping results. A variant called from Minimap2-aligned reads showed 100% concordance with the previous *CYP2D6* SNP calls. However, Lra’s performance was less reliable; among the 52 SNVs detected in the *CYP2D6* gene, three were false negatives and six were false positives, with five of the false positives being indels. In addition, 42130569 G > C (rs1080997) was incorrectly called 42130569insC in Lra aligned reads.

Moreover, we compared three widely used variant calling tools: Clair3 [27], NanoCaller [28], and PEPPER-Margin-DeepVariant [26]. PEPPER and Clair3 showed 100% concordance in SNV detections. However, PEPPER works well in lower read depth (~ 70x) and requires downsampling. Therefore, PEPPER was not applied to our study because the accurate integration of read count was crucial to answering our research question. NanoCaller and Clair3 were similar in SNPs detection. However, NanoCaller showed lower potential in detecting indels. For example, NanoCaller failed to detect two indel variants, rs774671100 and rs35742686, which are core variants for the CYP2D6*3 and *15 alleles, which cause a frameshift and loss of gene function. Overall, our analysis showed that Minimap2 for mapping and Clair3 for variant calling had the highest sensitivity and positive predictive value.

### Genetic diversity of *CYP2D6*, *CYP2D7* and *CYP2D8* genes

Variant calling for all samples was conducted twice: once using reads generated with triplex PCR, which were relatively short (~ 6.3–6.6Kb), and second using overlapping reads that covered the entire *CYP2D6-2D7-2D8* locus (entire regions ~30.2Kb). The variants called across the entire region were used to assess the probability of read misalignment across homologous genes and to measure the accuracy of variant calling from reads generated by triplex PCR. There was complete concordance (100%) between the variants called by the two methods. Additionally, successfully amplifying the entire locus allowed haplotype assignment across the region. A total of 52 SNVs (49 SNPs, two deletions, and one insertion) were detected for the *CYP2D6* gene across the 11 individuals. All of these were *CYP2D6*-defining variants. Twelve out of 52 detected *CYP2D6* variants had nucleotides corresponding to *CYP2D7* and *CYP2D8* reference nucleotides.

The successful amplification of the entire *CYP2D* locus has also enabled the phased variant analysis of the two pseudogenes, in addition to the *CYP2D6* gene. Our analysis identified 21 and 17 unique haplotypes for the *CYP2D7* and *CYP2D8* genes, respectively. These haplotypes harbour 108 SNVs (98 SNPs, eight deletions, and two insertions) for *CYP2D7* and 31 SNVs (29 SNPs and two insertions) for *CYP2D8*. Of these, 37 and 13 SNVs were in exons for the *CYP2D7* and *CYP2D8* genes, respectively. Of the variants found in *CYP2D7*, 57 had nucleotides corresponding to *CYP2D6* reference nucleotides, and 22 have been annotated as SNV in the *CYP2D6* gene, but two have different alternative variants. Similarly, 14 variants in the *CYP2D8* gene had nucleotides corresponding to *CYP2D6* reference nucleotides, and three have been annotated as SNVs in the *CYP2D6* gene, but one variant has an alternative variant. Moreover, 36 variants found within the pseudogenes have also been annotated as functional variants in the *CYP2D6* gene. Comparing the three *CYP2D* genes, *CYP2D7* was the most diverse, whereas *CYP2D8* was the least diverse among the *CYP2D* genes. The level of polymorphism among the three genes, especially between *CYP2D6* and *CYP2D7*, was relatively similar, indicating that when one gene was more diverse in an individual, the other was also diverse in that individual.

### *CYP2D6* gene copy number variations (CNVs)

**Fig. 2:**
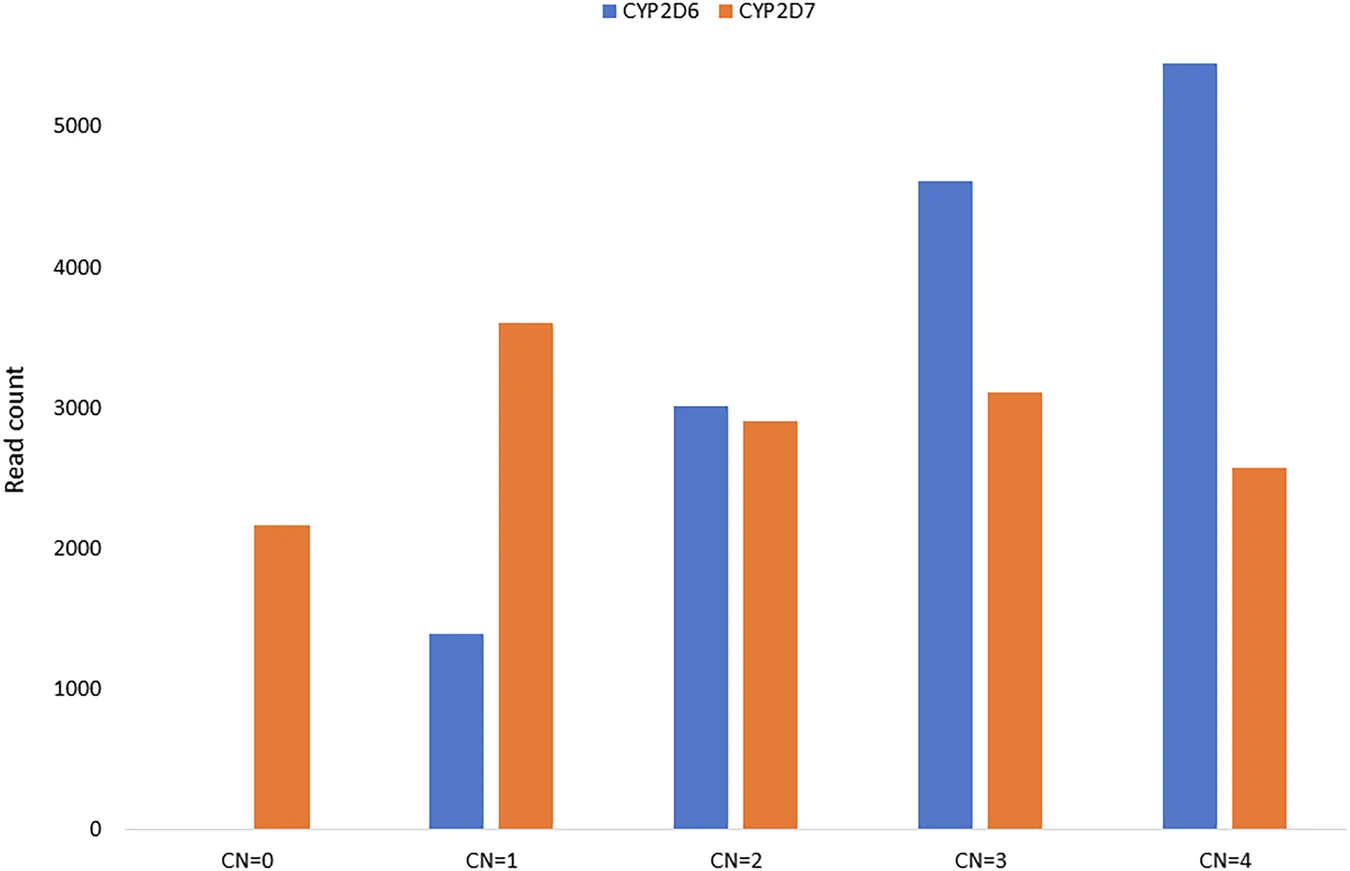
Average read counts mapped to *CYP2D6* and *CYP2D7* genes for individuals with *CYP2D6* CNV. CN indicates the copy number of the *CYP2D6* gene, which includes whole gene deletion (CN = 0), partial deletion (CN = 1), wild or without CNV (CN = 2), partial duplication (CN = 3), and full duplications (CN = 4).

The presence of *CYP2D6* CNV was determined by comparing the read depth with the *CYP2D7* gene (reference gene) and the allele frequency (AF) ratio between the alleles of *CYP2D6* variants. This is under the assumption that a 1:1 ratio indicates wild-type genes, and an uneven ratio suggests CNVs. A total of 10 individuals were included in the *CYP2D6* CNV analysis, and these individuals carried whole gene deletion (CN = 0), partial deletion (CN = 1), normal (CN = 2), partial duplication (CN = 3), and full duplication (CN = 4) (Table 3). The number of reads aligned to *CYP2D7* was consistent for both individuals with and without CNV (Fig. 2). However, the number of reads aligned to *CYP2D6* was lower for individuals with gene deletion and higher for individuals with gene duplication than in individuals without CNV (Fig. 2).

**Table 3:** *CYP2D6* copy number of variation (CNV), *CYP2D6*: *CYP2D7* read depth ratio and allele frequency ratio.

ID	*CYP2D6* CNV	Copy number (CN)	*CYP2D6*:*CYP2D7* read depth ratio	*CYP2D6* allele frequency (AF) ratio
NA10005	Wild	CN = 2	01:01	0.46
NA10181	Wild	CN = 2	1.39:1	0.45
NA19789	Wild	CN = 2	01:01	na
NA23877	Wild	CN = 2	01:01	0.43
NA08873	Deletion	CN = 1	01:01.6	na
NA19317	Deletion	CN = 0	01:00	na
NA07439	Duplication	CN = 3	1.4:1	0.3
NA17244	Duplication	CN = 4	02:01	0.5
NA17454	Duplication	CN = 4	02:01	0.5
NA18526	Duplication	CN = 3	1.6:1	0.4

*AF= na (not available) is for individuals without a variant or when all the variants were homozygous and unable to compare AF.

**Fig. 3:**
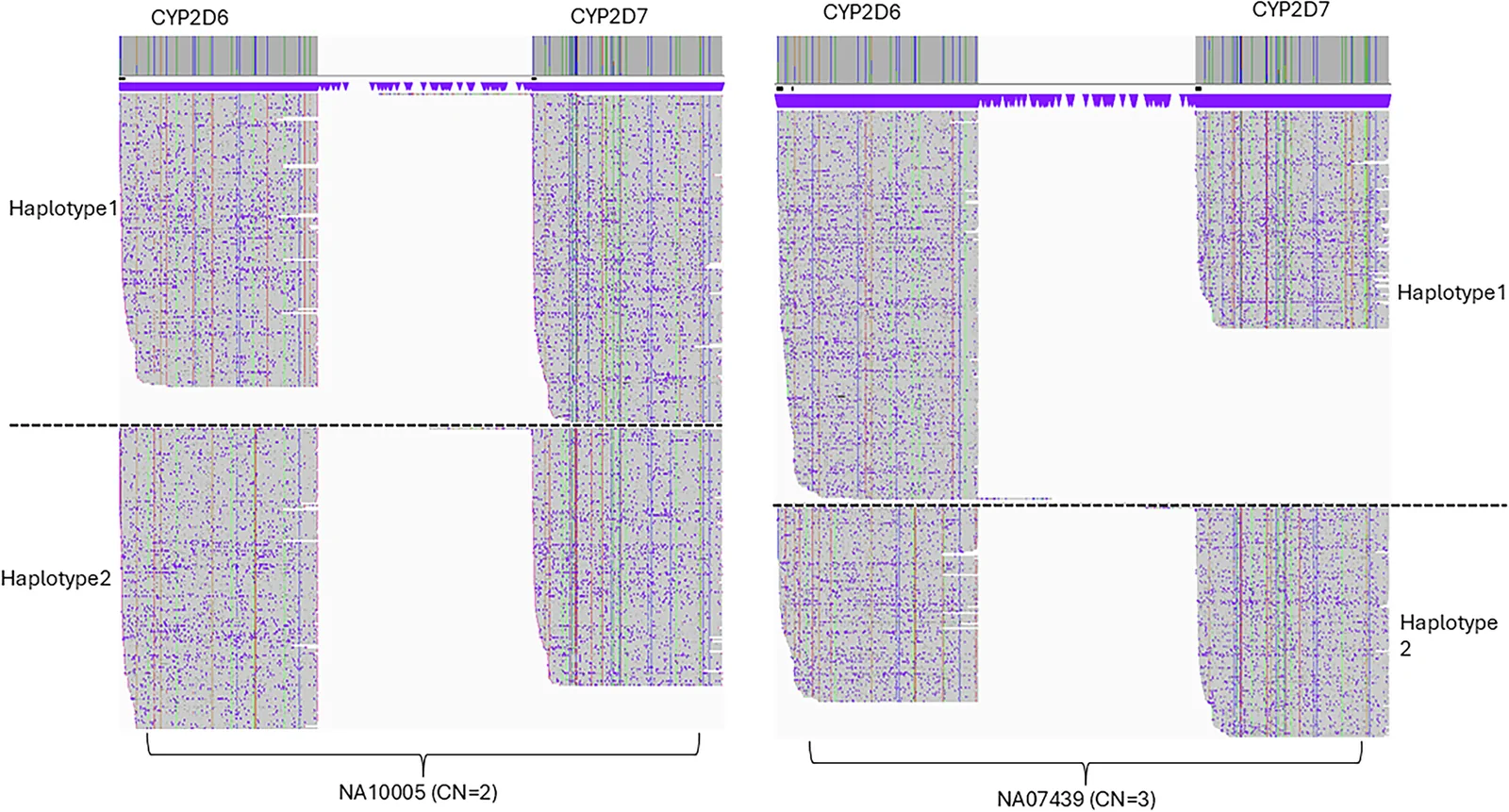
Phased reads aligned to *CYP2D6* and *CYP2D7* genes of individuals without CNV (NA10005) and with CNV (NA07439).

The read-ratio analysis between *CYP2D6* and *CYP2D7* consistent with *CYP2D6* CNV status. For example, the read count ratios for *CYP2D6* and *CYP2D7* were ~1:1 in individuals without CNV, 1:1.6 for partial deletion, and 1:0 for whole gene deletion (Fig. 2). For individuals carrying *CYP2D6* duplication, the ratio was either ~1.5:1 for CN = 3 or 2:1 for CN = 4. When individuals carry partial duplication (CN = 3), the duplicated alleles were determined based on overrepresented phased variants, i.e., by comparing the AF ratio of the reference and alternative of *CYP2D6* heterozygous variants (Table 3). For example, the AF ratio for individual NA07439 (CN = 3) was 0.3, indicating uneven read distribution and showing which haplotype of the gene was duplicated. Furthermore, the *CYP2D6* CNV was confirmed by visualizing phased long reads in IGV, which clearly indicated the CNV and the duplicated allele (Fig. 3). Overall, the read count and the AF ratio combinations can predict the CNV status of the *CYP2D6* gene.

## Discussion

Despite *CYP2D6* being the only functional gene, it is essential to understand the genetic variation of the two *CYP2D* pseudogenes to interpret the unusual genotypes or no calls caused by interfering variation in these pseudogenes [18]. In the current study, the variants detected for the *CYP2D6* gene were 100% concordant with the previous studies [30–32]. However, 23% of *CYP2D6* variants had nucleotides corresponding to *CYP2D7* and *CYP2D8* reference nucleotides. In addition, 57% (79/139) of the SNVs detected in the pseudo genes had nucleotides corresponding to *CYP2D6* reference nucleotides. These might contribute to misalignments and false-negative or false-positive genotyping results when using microarray- or NGS-based techniques to genotype the *CYP2D6* gene.

Furthermore, 36 variants found within the pseudogenes have also been annotated as functional variants in the *CYP2D6* gene. For example, rs3021082 (A > G) and rs1187217541 (dupC), located in exon 5 of the *CYP2D7* gene, match to rs199535154 (A > G) and rs72549354 (dupC), located in exon 4 of the CYP2D6 gene, respectively. The latter two variants are missense (rs199535154) and frameshift (rs72549354) variants, which cause loss of gene function and are core variants for CYP2D6*20 (CPIC no function). CYP2D6*20 includes two additional variants, such as rs1135840 (C > G) and rs16947 (G > A), which are also core variants for CYP2D6*2 (normal function). Therefore, this can lead to false-positive CYP2D6*20 calls for individuals carrying CYP2D6*2 when using conventional genotyping techniques. This is due to the sequence homology, which allows primer binding and amplification from the *CYP2D6* and *CYP2D7* genes, leading to genotyping errors. Similar findings have been reported for CYP2D6*15 and *35 alleles, where variants in the *CYP2D7* gene match the corresponding *CYP2D6* variants sequence [18]. Riffel et al. [18] reported that approximately 79% (55/70) of the samples genotyped as CYP2D6*15 were false positives and concluded that it is almost impossible to design a *CYP2D6*-specific genotyping assay given the sequence similarity between the two genes. Overall, the *CYP2D6* gene shares many identical variants with *CYP2D7* and *CYP2D8*, suggesting a high risk of genotyping errors and incorrect phenotype assignment. The genotyping community should expect a higher risk of genotyping errors for *CYP2D6* when using conventional genotyping techniques due to misalignment with paralogous pseudogenes. This can be more elaborated in individuals with SV/CNV or from populations that have not been well characterised [13].

The most commonly used method to determine *CYP2D6* CNV is long-range PCR (XL-PCR) targeting CYP-REP regions flanking the *CYP2D6* gene [33, 34]. This method detects the presence or absence of CNV but cannot distinguish between partial deletions or duplications. Other methods include Real-Time PCR Signal [34], quantification-based Pyrosequencing genotyping [35], imputation algorithms [36], high-resolution melting of PCR [37], and quantitative real-time PCR or digital droplet PCR [38]. These methods are based on the relative quantification of the PCR products relative to the housekeeping gene, in which the number of PCR products reflects the CNV status of the *CYP2D6* gene. However, these methods are laborious, unreliable, and require additional instruments and sample preparations. In addition, most of these methods do not discriminate against partial *CYP2D6* deletions/duplications. Rubben et al. [39] and Turner et al. [12] have reported a novel method utilising PCR-free CRISPR-Cas9 to target the complete *CYP2D6-CYP2D7-CYP2D8* loci using long-read sequencing, achieving lengths of up to 52 kb, including CNVs. However, the complexity of sample preparation and data analysis can limit its clinical applications.

In the current study, we followed the concept of quantifying the PCR products, but we targeted the whole genes (6.3–6.6Kb), sequenced using LRS, and quantified the sequence reads. We developed a sequencing-based method to detect *CYP2D6* CNVs using *CYP2D7* as a reference gene. We found that the *CYP2D6*:*CYP2D7* gene read count ratio was proportional to the *CYP2D6* CNV status. The number of reads aligned to the *CYP2D6* gene was twice or 1.5 times higher than the reads aligned to the *CYP2D7* gene for individuals with full (CN = 4) or partial *CYP2D6* duplication (CN = 3) (Fig. 3). The pattern was similar for *CYP2D6* deletion: approximately zero reads for whole *CYP2D6* gene deletion (CN = 0), and 2/3 of the reads aligned to *CYP2D7* for partial *CYP2D6* gene deletion (CN = 1). In addition, we used allele frequency (AF) imbalance to identify the duplicated allele in individuals with partial duplications. Sequencing the entire *CYP2D6* gene (6.6 kb) without fragmentation enables us to detect the overrepresented haplotype accurately.

## Conclusion

This is the first study to report the application of an amplicon-based LRS method to detect *CYP2D6* gene SNVs and CNVs, and to characterise the two homologous pseudogenes simultaneously. Here, we provided a proof-of-concept that the combination of the read count ratio between the two paralogous genes and AF imbalance accurately detects the *CYP2D6* gene CNV. We also characterise the SNV of two highly homologous pseudogenes and found that more than half of the variants in these pseudogenes had nucleotides corresponding to *CYP2D6* reference nucleotides. This approach is relatively rapid and shows potential to be easily integrated into clinical testing routines. Additionally, it has the potential to reduce overall genotyping time and costs by allowing simultaneous detection of SNVs and CNVs. Targeted Nanopore sequencing presents a lower initial investment and a clinically relevant turnaround time, rendering it a promising option for genotyping complex genes.

Our study found substantial sequence variations in the three *CYP2D* genes and provided compelling evidence for the identification of CNVs within this complex genomic region. However, due to the limited sample size, these findings should be interpreted with caution. To validate these observations and better evaluate the potential clinical utility of this approach, further studies involving larger and more diverse cohorts, including underrepresented populations, are needed. It is worth noting that the CNV nature of the reference gene (*CYP2D7*) used in the current study is not well documented. This limitation may affect the interpretation of CNV estimates. Therefore, future studies should consider using alternative reference genes with well-established copy-number stability to enable more robust analyses and support clinical validation. Furthermore, there are significant variations among the currently available *CYP2D6* alignment, annotation, variant, and star-allele-calling tools. Therefore, further studies should also focus on developing novel tools that can handle long, continuous sequence reads with complex structural variation.

## Data Availability

Data are available with the corresponding author upon request.
